# A Mighty Small Heart: The Cardiac Proteome of Adult *Drosophila melanogaster*

**DOI:** 10.1371/journal.pone.0018497

**Published:** 2011-04-25

**Authors:** Anthony Cammarato, Christian H. Ahrens, Nakissa N. Alayari, Ermir Qeli, Jasma Rucker, Mary C. Reedy, Christian M. Zmasek, Marjan Gucek, Robert N. Cole, Jennifer E. Van Eyk, Rolf Bodmer, Brian O'Rourke, Sanford I. Bernstein, D. Brian Foster

**Affiliations:** 1 Development and Aging Program, NASCR Center, Sanford-Burnham Medical Research Institute, La Jolla, California, United States of America; 2 Department of Biology and the Heart Institute, San Diego State University, San Diego, California, United States of America; 3 Quantitative Model Organism Proteomics, Institute of Molecular Life Sciences, University of Zurich, Zurich, Switzerland; 4 Division of Cardiology, Institute of Molecular Cardiobiology, Johns Hopkins University School of Medicine, Baltimore, Maryland, United States of America; 5 Department of Cell Biology, Duke University School of Medicine, Durham, North Carolina, United States of America; 6 Bioinformatics and Systems Biology Program, Sanford-Burnham Medical Research Institute, La Jolla, California, United States of America; 7 Johns Hopkins Proteomics Core, Johns Hopkins University School of Medicine, Baltimore, Maryland, United States of America; 8 Division of Cardiology, Departments of Biological Chemistry and Biomedical Engineering, Johns Hopkins Bayview Medical Center, Baltimore, Maryland, United States of America; University of Minnesota, United States of America

## Abstract

*Drosophila melanogaster* is emerging as a powerful model system for the study of cardiac disease. Establishing peptide and protein maps of the *Drosophila* heart is central to implementation of protein network studies that will allow us to assess the hallmarks of *Drosophila* heart pathogenesis and gauge the degree of conservation with human disease mechanisms on a systems level. Using a gel-LC-MS/MS approach, we identified 1228 protein clusters from 145 dissected adult fly hearts. Contractile, cytostructural and mitochondrial proteins were most abundant consistent with electron micrographs of the *Drosophila* cardiac tube. Functional/Ontological enrichment analysis further showed that proteins involved in glycolysis, Ca^2+^-binding, redox, and G-protein signaling, among other processes, are also over-represented. Comparison with a mouse heart proteome revealed conservation at the level of molecular function, biological processes and cellular components. The subsisting peptidome encompassed 5169 distinct heart-associated peptides, of which 1293 (25%) had not been identified in a recent *Drosophila* peptide compendium. PeptideClassifier analysis was further used to map peptides to specific gene-models. 1872 peptides provide valuable information about protein isoform groups whereas a further 3112 uniquely identify specific protein isoforms and may be used as a heart-associated peptide resource for quantitative proteomic approaches based on multiple-reaction monitoring. In summary, identification of excitation-contraction protein landmarks, orthologues of proteins associated with cardiovascular defects, and conservation of protein ontologies, provides testimony to the heart-like character of the *Drosophila* cardiac tube and to the utility of proteomics as a complement to the power of genetics in this growing model of human heart disease.

## Introduction

**Figure 1:**
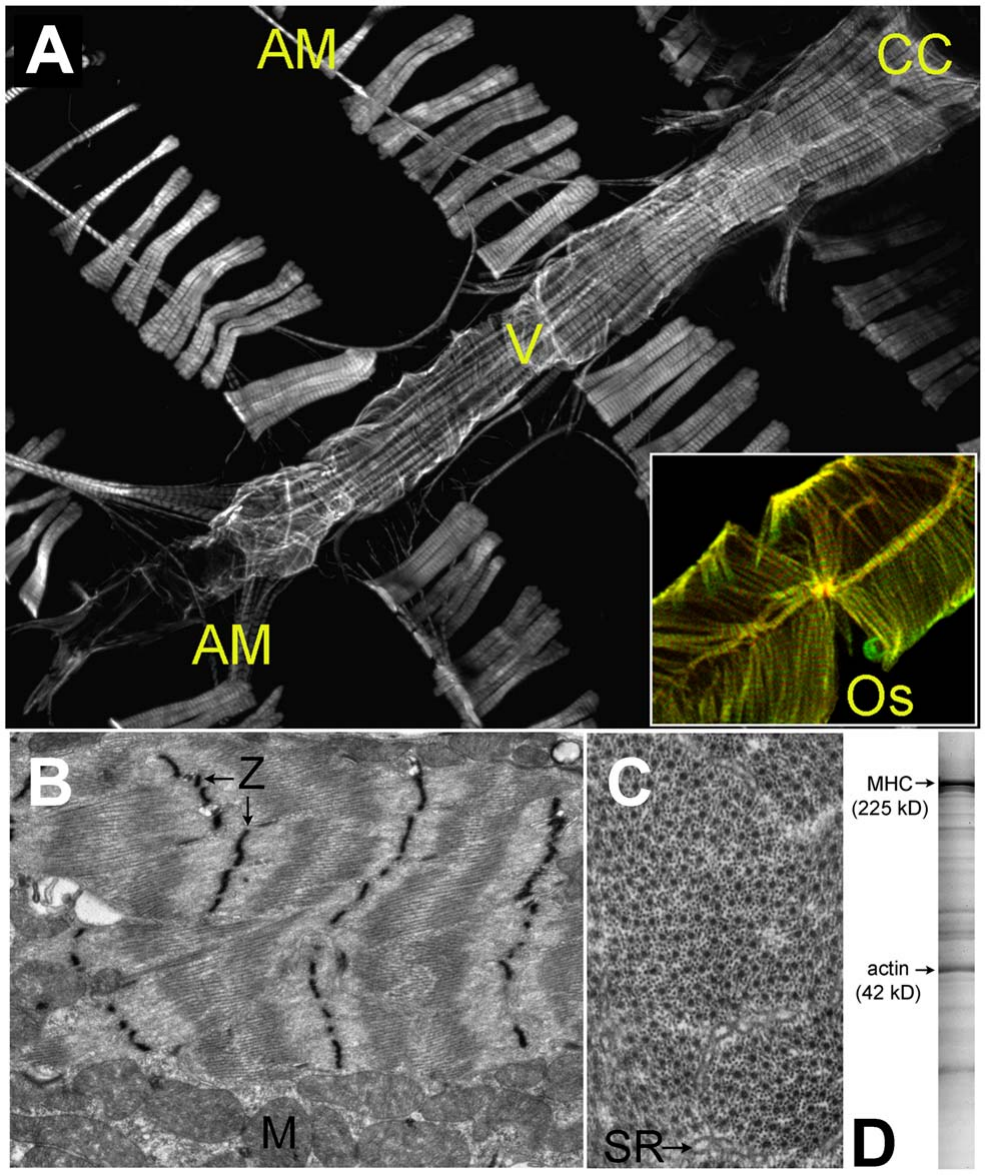
The Cardiac Tube *of Drosophila melanogaster*. Panel A. TRITC-Phalloidin labeled wild-type *Drosophila* heart tube and associated structures (10× magnification). CC = conical chamber; AM = alary muscle; v = internal valve; Os = ostia in flow tract. Inset: luminal surface of TRITC-Phalloidin-labeled myosin-GFP-expressing heart (20× magnification). Ostia inflow tracts and the striated alternating myosin and actin myofilament bands are clearly resolved. Panel B. Electron micrograph of a longitudinal section through the conical chamber reveals the contractile myofibrils and mitochondria (M)(3,800×). Densely stained Z-bands (Z) demarcate individual sarcomeres and bisect the I-bands. Centrally-located A-bands are also apparent. Panel C. Cross-section through cardiac myofibrils of the conical chamber (10,500×). Individual thick filaments are surrounded by 9–11 thin filaments. Regions of sarcoplasmic reticulum (SR) can also be resolved. Panel D. 10% Coomassie-stained polyacrylamide gel from 30 *Drosophila* heart tubes. Sarcomeric myosin heavy chain (MHC) and actin are highlighted for reference.

Long valued as a prime model of cardiac development, the utility of *Drosophila melanogaster* for the study of cardiac pathogenesis and pathophysiology is growing rapidly [1], [2], [3], driven by the development of new research tools and methods [4], [5], [6]. Adult *Drosophila* possess an open circulatory system consisting, in part, of a dorsal vessel (Fig. 1) which is differentiated into an abdominally-located ∼1 mm long pulsatile heart tube and an anterior aorta that extends through the thorax and into the head [7]. The prospect of combining quantitative proteomics of the cardiac tube with the power of *Drosophila* genetics promises to provide novel insights into the mechanisms of human heart disease.

Two significant roadblocks to widespread adoption of *Drosophila* as a model system for the study of heart disease need to be overcome. The first is technical. The small size of the *Drosophila* cardiac tube presents a challenge that is being addressed by the development of adequate dissection protocols [8] and imaging methods [8], [9]. Application of proteomic techniques presents its own unique challenges, not the least of which is collecting sufficient protein for study.

A second impediment is the diminishing, yet persistent, view that the *Drosophila* cardiac tube is not a “true heart” and that its study may yield few insights translatable to human disease mechanisms. However, recently published work would suggest otherwise [1], [10], [11], [12], [13]. The pathological effects of fly and human mutant protein isoforms, expressed in the *Drosophila* cardiac tube, have successfully predicted causal-genes that are both involved in, and recapitulate the phenotypes of, specific human cardiomyopathies [1], [10], [13], [14]. Thus, unbiased, high-throughput mutagenesis screens in flies followed by cardiac phenotyping, can be integrated for rapid gene discovery and novel network reconstruction to greatly facilitate cardiac systems biology and to elucidate pathogenic mechanisms of human cardiac disease [15].

A critical component essential for further exploiting the power of *Drosophila* in cardiac systems biology is a comprehensive record of the protein constituents of the *Drosophila* heart and associated cardiac tissues. Here we establish, for the first time, a peptide and protein compendium of the adult *Drosophila* heart, and assess the extent of protein conservation with a mammalian model, further solidifying the relevance of the *Drosophila* model as a surrogate for the study of human heart disease.

## Methods

### Dissection of the Cardiac Tube

*yw* wild-type *Drosophila melanogaster* were raised on a standard yeast-agar medium at room temperature. The cardiac tubes of 145 male and female adult flies, ranging from 1 to 7 weeks of age, were dissected and exposed according to Vogler and Ocorr (2009) [8]. Briefly, flies were anesthetized and the heads, ventral thoraces, and ventral abdominal cuticles were removed, exposing the heart tubes. All internal organs and abdominal fat were carefully removed leaving the heart and associated cardiac tissues. Dissections were performed under oxygenated artificial hemolymph at room temperature and all heart tubes were examined for activity prior to removal. The conical chambers (Figure 1A) were grasped with forceps and the hearts were gently removed and quickly transferred to an Eppendorf tube containing 1.5 ml of artificial hemolymph on ice. The hearts continued to beat immediately following their removal. The tissue was pelleted (10,000 rpm) and washed three times quickly in distilled deionized water at 4°C. The sample was then lyophilized and the cardiac tubes dehydrated and stored at −80°C.

### Fluorescence & Electron Microscopy

Fluorescence microscopy was performed as detailed by Alayari *et al.* [9]. Briefly, wild-type (*yw*) *Drosophila* hearts or hearts expressing myosin-GFP (obtained from http://flytrap.med.yale.edu) were labeled with TRITC-phalloidin and imaged with a Zeiss Imager Z1 fluorescent microscope equipped with an Apotome sliding module at 10 and 20× magnification. Electron microscopy was performed with a Philips CM 420 electron microscope essentially as described by Wolf *et al.* (2006) [13], however, prior to fixation with Karnovsky fixative (3% formaldehyde/3% glutaraldehyde in 0.1 M Na-cacodylate buffer, pH 7.35), the cardiac tubes were exposed and dissected free of extraneous debris as described by Vogler and Ocorr (2009) [8]. Electron micrographs of semithin sections through the conical chamber were acquired at 3,800× and 10,500× magnification.

### Sample Preparation and Mass Spectrometry

The washed and lyophilized hearts were homogenized in reducing SDS-sample buffer (NUPAGE, Invitrogen) containing 6M urea. Thirty (30) heart tubes provide sufficient protein to detect and resolve the major protein constituents via denaturing SDS-PAGE and Colloidal Coomassie Blue staining (Simply Blue, Invitrogen). The reported dataset was obtained from homogenization of 145 hearts (∼20 µg protein) and further processing with a gel-LC-MS/MS proteomics approach. A single gel lane was cut into 13 tranches. Each tranche was subjected to in-gel trypsinolysis and peptide extraction by the method of Shevchenko *et al.* [16]. Extracted peptides were subjected to 4-replicate runs to LC-MS/MS on a LTQ ion-trap mass spectrometer (Thermo). Details regarding chromatography, apparatus and instrumentation settings are found in Methods S1.

### Database Searching

Tandem mass spectra were extracted by Bioworks 3.3. All MS/MS samples were analyzed using Mascot (Matrix Science, London, UK; version Mascot) and X!Tandem (www.thegpm.org; version 2007.01.01.1). Mascot was set up to search a database of *D. melanogaster* reference protein sequences (Refseq) downloaded from the National Center for Biotechnology Information (NCBI) in FASTA format. The database was current as of 09/24/2008 and contained 20735 entries. X!Tandem searches were conducted using the same database. Searches were conducted using trypsin as the digesting enzyme. Mascot and X!Tandem were searched with a fragment-ion mass tolerance of 0.80 Da and a parent-ion tolerance of 1.5 Da. Carbamidomethylation of cysteine was specified in Mascot and X!Tandem as a fixed modification. Oxidation of methionine was allowed as a variable modification.

### Criteria for Protein Identification

Scaffold (version 2.02.04; Proteome Software Inc., Portland, OR) was used to validate MS/MS based peptide and protein identifications. Peptide identifications were provisionally accepted if they had a >90.0% probability, as specified by Scaffold's implementation of the Peptide Prophet algorithm [17]. Proteins that contained similar peptides and could not be differentiated based on MS/MS analysis alone were grouped to satisfy the principles of parsimony [18]. To maximize the sensitivity of discovery, given limited starting material (145 hearts, ≈20 ìg of protein), identifications were accepted provisionally if they contained at least 1 statistically-validated unique peptide from 1 assigned spectrum. Recent studies have demonstrated the value of single hit protein identifications [19], [20], as long as care is taken to remove potential false-positive identifications. Specifically, proteins identified on the basis of single spectrum/peptide matches were inspected manually and accepted only if they: 1) were well fragmented, displaying contiguous b- and y-ion stretches, 2) showed complementary b- and y-ions, 3) were scored at 90% probability by Peptide Prophet, and either 4) matched reference spectra from the dataset of Brunner *et al.* archived at the National Institute of Standards and Technologies (Tables S3, S4, S5, S6, S7, S8), or 5) conformed with well-established peptide fragmentation biases [21](Table S9).

### Bioinformatic Analysis

Ontological protein classification and clustering of the *Drosophila* cardiac dataset were conducted using the Database for the Annotation, Visualization and Integration of Data (DAVID) (http://david.abcc.ncifcrf.gov/) and ProteinCenter (Proxeon). Ontological and functional domain comparisons between *Drosophila* and mouse proteomic datasets were conducted using Ontologizer 2.0- a multifunctional software tool for GO term enrichment analysis and data exploration [22]. A discussion of the limitations and provisos associated with such comparisons can be found in Methods S1.

## Results

### Drosophila Cardiac Tubes Used In This Study

The adult *Drosophila melanogaster* heart tube extends medially from the first through the sixth abdominal segment close to the dorsal body wall [7] (Figure 1A). It consists of a single muscular layer of circular contractile cardiomyocytes that join together to create the heart wall, three pairs of opposing “spongy” internal valve cells that project into the lumen from the wall, and five pairs of ostial inflow tracts [5], [7], [23]. The anterior conical chamber, the most pronounced muscular region of the heart tube, is ∼120 µm wide and tapers gradually through the first two abdominal segments. The remainder of the heart tube is roughly 50 µm in diameter along its length. In addition to the cardiomyocytes highlighted in figure 1, the cardiac tube also closely associates with a ventral longitudinal muscle layer, pericardial cells, extracellular matrix and is innervated by neurons (not shown). The dissected cardiac tubes used in subsequent proteomic studies contained all aforementioned structures.

### Overview of Proteins from Adult Drosophila Cardiac Tube

Data collected from 145 *Drosophila* hearts resolved by 1D-gel electrophoresis initially yielded 1520 protein candidates that met the minimal statistical threshold for provisional acceptance (one peptide with >90% probability). 766 proteins were identified by at least 2 unique high-quality peptides (>90% peptide probability) with a protein identification probability >99.9%, on the basis of Scaffold's implementation of the empirical Bayesian algorithms, Peptide Prophet and Protein Prophet, respectively [17]. To extend the heart proteome coverage to lower abundance and shorter proteins [19], we also considered proteins identified by a single unique peptide as described in the methods section. The merits of including 1-hit proteins in datasets have been addressed recently [19], [20]. To minimize false discovery, single-peptide hits among the 1520 provisional proteins were filtered using a stringent multistep cross-validation process as outlined in Methods S1. Three-step evaluation removed 292 single-hit protein candidates, yielding a final complement of 1228 proteins clusters, identified by 5169 unique peptide matches from 29862 assigned spectra (Tables S1, S2).

### Specificity of the Drosophila Cardiac Proteome:

#### 1. Comparison with the Extensive Drosophila Proteome of Brunner et al [24]

**Figure 2:**
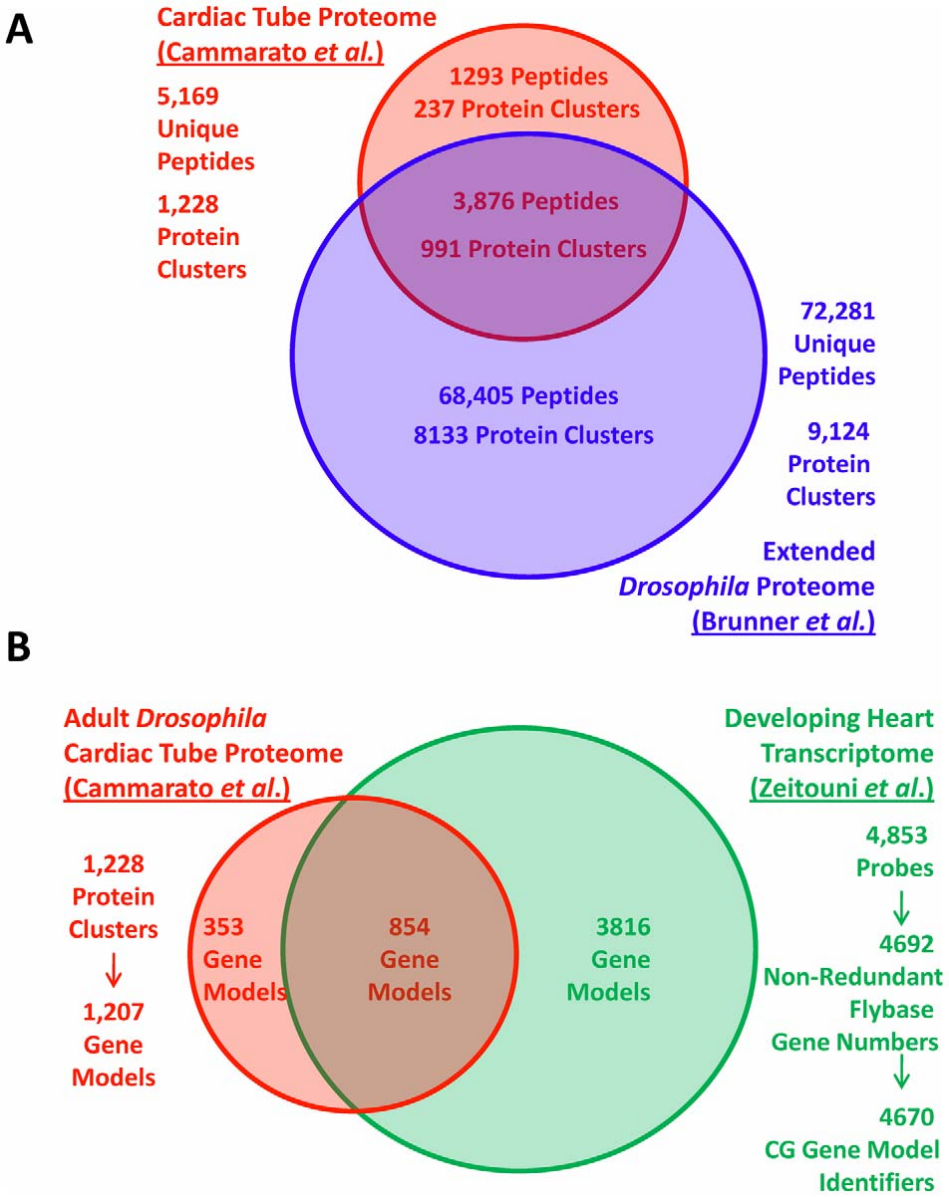
Specificity of the *Drosophila* Cardiac Proteome. Panel A. The cardiac tube proteome was compared with the extensive *Drosophila* proteome of Brunner *et al* [24]. To minimize complications arising from the use of different databases (Refseq vs. BDGP3.2), comparison at the level of peptides is preferred. 1293 peptides, or approximately 25% of those identified in this study, were uniquely detected in our heart dataset and ultimately mapped to 237 protein clusters that were novel to the cardiac tube dataset. Panel B. The cardiac tube proteome was cross-referenced with the developing heart transcriptome of Zeitouni *et al* [25]. Protein and transcript datasets were mapped onto CG gene models to facilitate comparison (see Methods S1).

To assess the tissue specificity of our proteome we compared our dataset to the landmark work of Brunner *et al* [24] (Figure 2A), an extensive *Drosophila* peptidome/proteome compiled from a variety of *Drosophila* cell lines and body segments. Of the 5169 unique peptides we observed (Table S1) 1293 were not found among the 72281 detected previously and are, therefore, novel to the heart tube proteome. Importantly, only 25 peptides of the 5169 peptide matches found from searching the Refseq database were not present in BDGP3.2 database used previously [24]. Therefore, the bulk of the novel identified peptides do not arise simply from the use of different databases for analysis, but rather, stem from the use of isolated *Drosophila* cardiac tubes, which had not been analyzed in the Brunner *et al.* study.

The 1293 novel peptides mapped to 237 protein clusters (19%) that were unique to our cardiac tube proteome dataset (Table S10). Ontological enrichment analysis of the unique proteins showed that metabolic, mitochondrial and muscle-related ontologies are more prominent than would be expected by chance (Benjamini-corrected p<0.05; Figure 2B). Enriched biological processes included carbohydrate metabolism (GO:0005975) muscle contraction (GO:0006936) and muscle system (GO:0003012). Among enriched molecular functions, ion pumping ATPases (GO:0042623), and oxidoreductase activity (GO:0016491), likewise figure prominently (see Table S10).

#### 2. Comparison with a Drosophila Cardiac Transcriptome

As an independent assessment of specificity, we compared our dataset with transcriptomic data from a published study of early *Drosophila* heart development [25]. In their time-course study, covering 8 time points from 21 to 48 hours after puparium formation, Zeitouni et al. had found about 4800 gene models to be consistently expressed above background levels (for details, see Methods S1). 854 of our 1207 gene models (encoding the 1228 proteins) were observed in both proteomic and trancriptomic datasets (71%; Figure 2B). Another 285 gene models encoding proteins found in the adult cardiac tube (24%) were present on the microarray but not expressed above the chosen threshold. These may represent gene models that are more prominently expressed during later stages of heart development. Taken together, comparison with the broader extensive *Drosophila* proteome and the transcriptome of early heart development demonstrates that dissection has successfully led to a cardiac tube-enriched proteome.

### Abundant Protein Classes in the Drosophila Cardiac Proteome

**Figure 3:**
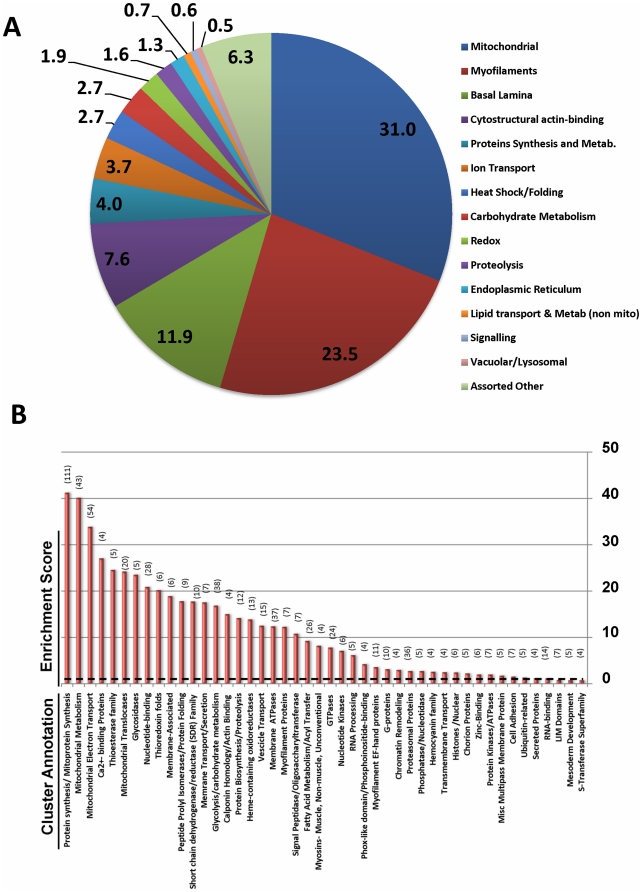
Annotation and Classification of the *Drosophila* Cardiac Proteome. Panel A *By abundance*: Using the number of assigned spectra as a measure of relative protein abundance, the top 245 proteins (20%) were annotated manually with information from NCBI and Flybase. Total assigned spectra within a group are expressed as a percentage of the total number of spectra assigned to the top 245 proteins. The chart provides a measure of the relative abundance of proteins that comprise each group. Panel B. *By clustering & enrichment of gene-ontology terms*: 928 proteins for which functional annotation was available among the 1228 proteins were subjected to functional clustering and enrichment analysis using the Functional Classification tool at the DAVID knowledgebase. Approximately 600 proteins were grouped into 47 functional classes based on the similarity of their gene-annotations. The annotation clusters are ranked by their *enrichment score* (−log(p-value)). The number of proteins per cluster is indicated in parentheses. A score of >2 (**—**) denotes high probability that a class is enriched.

To get a qualitative assessment of the relative abundance of identified proteins, we examined the number of total assigned spectra for each protein. Spectral assignments followed an 80/20 distribution, i.e. 20% of identified proteins (245) accounted for nearly 80% (78.8%) of the total assigned spectra. Figure 3A shows these most abundant proteins categorized manually, guided by annotation terms available from NCBI and Flybase. Consistent with the electron micrographs of *Drosophila* cardiac muscles (Figures 1B, 1C) depicting alternating arrays of sarcomeres and mitochondria, the list is dominated by myofilament, cytostructural and mitochondrial proteins. Myosin heavy chain alone, owing to its abundance and high molecular weight, accounts for fully 10% of all assigned spectra. The most abundant myofilament and cytostructural proteins, together, account for 31% of assigned spectra among the top 245 proteins (23% and 8% respectively). Mitochondrial proteins were also among the most abundant. Spanning diverse functions including fatty-acid oxidation, tricarboxylic acid (TCA) cycle and oxidative phosphorylation, they also accounted for about 31% of the spectra. Proteins of the basal lamina that provide structural integrity of the cardiac tube, including several laminins and collagens, accounted for a further 12%. Other noteworthy classes include the proteins involved with protein synthesis, ion transport, heat-shock response and carbohydrate metabolism. Individual proteins within each group are shown in Table S11.

### Functional Annotation Enrichment Analysis

To determine which biological functions were over-represented in our cardiac-tube dataset, we undertook functional annotation and enrichment analysis using tools available from DAVID [26], [27]. By this ontological analysis, each entry was categorized according to their biological processes, cellular components, and molecular functions (Table S12). Functional clustering and enrichment analysis found that approximately 928 of the 1228 proteins could be classified into 47 functional categories. Figure 3B lists functional annotation clusters, ranked by degree of enrichment within the dataset. Notably, ribosomal and mitochondrial ribosomal functional annotations were particularly over-represented. Consistent with our assessments of protein abundance, functions commonly associated with mitochondria were also over-represented, commensurate with the high energy demands of this myofilament rich contractile tissue (Fig. 1B, 1C, 3A). Ca^2+^-binding proteins and proteins with thioredoxin-folds are highly enriched, attesting to the importance of Ca^2+^ handling and redox regulation in the *Drosophila* heart. Likewise, proteins involved in protein folding (chaperones and cyclophilins), glycolysis, and varied oxidoreductase enzymes figure prominently by this measure. Among signaling proteins, those of the low molecular weight ras-like GTPase superfamily are well represented, including ras, several rab proteins, rac1, rho1 and cdc42. Kinases identified include Ca-calmodulin dependent kinase II, casein kinase, integrin-linked kinase and pyruvate dehydrogenase kinase.

### The Drosophila Cardiac Proteome in Context

#### 1. Identification of Cardiac Proteins Essential for Fly Survival/Orthologs of Vertebrate Proteins Critical for Heart Function

We recently performed a genome-wide RNAi screen to identify conserved cardiac genes whose products are essential for *Drosophila* survival under conditions of stress [14]. Heart-restricted silencing of 498 genes significantly increased mortality when the flies were exposed to elevated temperatures. These gene candidates, when knocked down, likely result in severe cardiac functional abnormalities since the *Drosophila* heart can be dramatically altered and not necessarily initiate organismal death. Seventy-four (74) of these vital 498 genes (15%) had protein products detectable in our proteome. Furthermore, 73% of the 74 genes corresponded to orthologs found in the cardiovascular system of vertebrates (humans and/or mice; determined via the NextBio web-based platform (http://www.nextbio.com/b/nextbio.nb)) and, 40% have orthologs implicated in diverse cardiac related disorders including cardiomyopathy, myocardial infarction, cardiac arrest and heart failure (See Table S13).

#### 2. Comparison with the Mouse Heart Dataset

**Figure 4:**
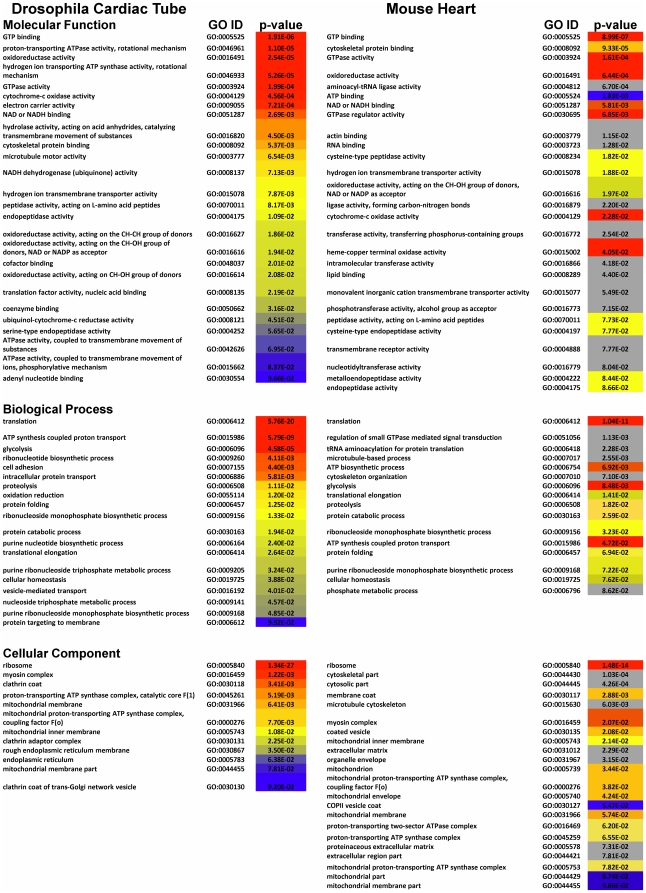
Comparison of the *Drosophila* and Mouse Heart Proteomes. Functional descriptions of protein domains (as defined by the Pfam database [42]) of the *Drosophila* cardiac proteome (left) and the mouse heart proteome (right) were subjected to Gene-ontology term enrichment analysis using Ontologizer 2.0 software [22] with the Topology-Elim algorithm [43] and Bonferroni correction. The table is laid out according to the three branches of ontology: Molecular Function, Biological Process or Cellular Component. Annotation terms within each section are listed in descending order of enrichment (lowest p-values at the top). Within each branch of ontology *Drosophila* terms are color coded from red (lowest p-value) to blue (highest p-values). These colors were mapped onto related ontological terms found in the mouse to highlight commonalities (colors) and differences (grey).

To assess the similarity between *Drosophila* and mammalian hearts, we compared the functional ontological profile of its proteome with that of a reference heart dataset from mouse (Figure 4). Analysis of the *Drosophila* cardiac proteome revealed 866 protein family (Pfam) domains. Of these, 706 (82%) were conserved in the mouse heart (Table S13). Comparison of gene-ontology annotations is summarized in Figure 4. Note the similarities between the *Drosophila* cardiac tube and the mouse heart at the level of cellular components, biological processes and molecular function (color-matched ontology terms). GTPase, oxidoreductase and other mitochondrial activities dominate the molecular function category in both the *Drosophila* cardiac tube and the mouse heart. Among biological processes, the enrichment of terms associated with protein synthesis (translation, translational elongation) in the *Drosophila* cardiac tube is mirrored in the mouse heart. Glycolysis and ATP synthetic processes are likewise highly-enriched in both species. Annotations of the cellular component category reaffirm what could be deduced from microscopy, namely that the cardiac tube and the mouse heart are dominated by myosin complexes (i.e. myofilaments) and mitochondria.

Our proteome is ontologically distinct from other smaller *Drosophila* proteomic datasets (e.g. *Drosophila* seminal fluid [28]; not shown) though it is similar to that of other mitochondria-rich organ sets, such as mouse liver [29]. The liver dataset, however, lacks the structural and myofilament protein complement -major components observed in mouse heart and the *Drosophila* cardiac tube. Note p-values in Figure 4 are not directly comparable between *Drosophila* and mouse datasets. They are indicative of enrichment within each dataset only. Finally, at this stage, it would be premature to attribute ontological differences (grey) to *bona fide* biological differences between the two species, since they might well stem from the differences in methodology and instrumentation bias (see Methods S1).

### Drosophila Cardiac Peptidome: A Resource for Multiple-Reaction-Monitoring Mass Spectrometry

One of the goals of quantitative proteomics is to robustly assess the levels and even the posttranslational status of any protein, or group of proteins, in the cell at a given time. Traditional shotgun proteomic strategies that identify as many proteins as possible from an enzymatic protein digest suffer the inevitable shortfall that low-abundance proteins are systematically under-sampled, making quantification difficult. One technology that promises to yield more sensitive protein maps has been used for the quantitative mass spectrometry of small molecule analytes for years. Known as single reaction monitoring (SRM) or multiple reaction monitoring (MRM) [30], it offers up to 100-fold greater sensitivity than shotgun proteomic approaches [31], and is uniquely suited for the targeted quantification of specific known peptides, based on characteristic chromatographic retention time, parent ion masses, and MS^2^-ion transitions. Yet, before MRM approaches can be successfully applied, certain criteria must be met. Firstly, of the tryptic peptides observable theoretically, only a fraction has physicochemical properties that favor detection by mass spectrometry. Secondly, even fewer peptides lend themselves to unambiguous protein identification. Types of peptides range from those that uniquely identify a specific protein isoform from a single gene, to those that arise from multiple unrelated proteins and therefore provide little protein/gene information.

To extract peptide-protein-gene model relationships, we subjected our 5169 unique cardiac tube peptides to a PeptideClassifier analysis according to Qeli and Ahrens [32], and ranked them in order of information content (Table S14). Of the 5169 peptides, 4984 were classified as either “proteotypic” or “information-rich”. Proteotypic peptides provide sufficient information to distinguish specific protein isoforms (Classes 1a, 1b, and 3a in Table 1). Information-rich peptides are common either to a subset, or to all protein isoforms encoded by a specific gene model (Classes 2a and 2b in Table 1). Particularly noteworthy are the 3112 proteotypic peptides among which 774 are newly-identified. Moreover, the remaining 2338 peptides identified previously in *Drosophila* cell lines and body segments [24], can now be assigned a role in the heart (Table 1).

**Table 1:** *Drosophila* Cardiac Peptidome.

Peptide Class^1^	Type of Peptide Evidence	# IdentifiedPeptides (%)	New Peptides
Class 1a	identifies one protein - one gene-model	2316 (44.8)	627
Class 1b	identifies one protein - encoded by isoforms differing in 5′ or 3′ UTR of one gene model	783 (15.1)	146
Class 2a	identifies a subset of protein isoforms	249 (4.8)	95
Class 2b	common to all protein isoforms encoded by a gene-model	1623 (31.4%)	377
Class 3a	identifies one protein from multiple gene-models	13 (0.3%)	1
Class 3b	peptides common to unrelated proteins	185 (3.6)	47

	Proteotypic Peptides^1^	Information-Rich Peptides^1^
	(Class 1a+Class 1b+Class 3a)	(Class 2a+Class 2b)
	Total: 3112	Total: 1872
	New: 774	New: 472

1 See Table S14.

Peptides identified in a shotgun proteomics experiment may be classified into 6 types on the basis of the information they impart about a gene model [32]. Proteotypic peptides are those that uniquely identify a specific protein isoform and may be encoded by multiple transcripts or multiple genes. Information-rich peptides are shared among protein isoforms arising from multiple transcripts or genes. Proteotypic peptides are particularly useful for the design of new high-sensitivity quantitative mass spectrometry methods based on multiple-reaction monitoring. New peptides were not previously in the *Drosophila* peptide compendium of Brunner *et al.* [24].

## Discussion

As *Drosophila melanogaster* is used increasingly as a model of heart disease, it behooves us to characterize its cardiac tube more fully, to better gauge the prospects and limitations of the system. Specifically, extending new insights from *Drosophila* to mammals demands a better understanding of the similarities between these hearts at a molecular level. Since the heart is, in part, the sum of its protein components, we undertook a proteomic approach.

Here, we have shown that the *Drosophila* cardiac proteome conforms, in terms of protein abundance and functional enrichment, to what one might expect given its ultrastructure by electron microscopy. But more importantly, the classes of proteins identified and enriched in our dataset mirror those found in a recently published comprehensive mouse heart proteome [33], which we used as a benchmark. Specifically, we note the similarities at the level of myofilament, structural and mitochondrial function. Moreover, *Drosophila* hearts share the redox buffering and Ca^2+^-handling proteins found in mammalian hearts. The comprehensive mouse heart proteome does include proteins under-represented in our dataset, however, notably kinases, certain ion channels and transmembrane receptors. We suspect this could well stem from differences in methodology, as the mouse hearts were fractionated into their subcellular components prior to analysis, which would favor identification of lower abundance proteins from the cytosol and membranes. Efforts are currently underway to identify under-represented *Drosophila* protein classes whose presence is predicted by preliminary cardiac transcriptome work (AC, NA, RB, SIB, DBF unpublished).

Genetic lesions expressed in the *Drosophila* cardiac tube are already revealing remarkable parallels with human heart disease. We recently demonstrated that knockdown of CCR4-Not components in *Drosophila* and in mice resulted in cardiomyopathy and heart failure and that a common *NOT3* SNP (rs36643) in humans correlates with altered cardiac QT intervals, a frequent cause of sudden cardiac death [14]. The degree of protein conservation observed here suggests that *Drosophila* heart studies will continue to provide a convenient extension of widely-used genetic mouse models of heart disease and provide translatable insights into human cardiac dysfunction. For example, identification of rac1 in the *Drosophila* heart suggests that it may provide a valuable model to complement mouse studies of rac1-mediated hypertrophy [34], [35].

The conservation between the *Drosophila* and mouse heart proteomes also bodes well for the implementation of systems biology approaches that include, among other techniques, computational modeling and proteomic network perturbation, to assess mechanistic commonalities between these model organisms. For instance, it will be important to test whether *Drosophila* cardiac function can be adequately described by the latest models of excitation-contraction coupling integrated with mitochondrial energetics (ECME) [36]. Our *Drosophila* proteome identifies and provides unique mass spectral signatures for many of the proteins integral to the model. These include major determinants of intracellular calcium regulation (voltage-gated Ca^2+^ channels, ryanodine receptors, SERCA, and PMCA), K^+^ and Na^+^ (Na^+^/K^+^ ATPase, Na^+^/Ca^2+^ exchanger), NADH production (TCA cycle proteins) and ATP levels (adenylate kinase, ATP synthase) (see Table S13).

Finally, compendia of experimentally observable isoform-specific peptides will be highly valued resources as we strive toward the goal of complete proteome coverage. Mass spectrometry techniques such as multiple-reaction monitoring are among those at the forefront of quantitative proteomic approaches [30] whose experimental design benefit greatly from observed peptide-spectrum matches. In this study, we have classified and ranked all 5169 identified peptides in order of the information they impart about a gene-model. Fully 3112 of these peptides were mapped to specific protein isoforms found in the cardiac tube. This peptide set will serve as an excellent complement to current proteotypic peptide prediction algorithms [37], [38] as well as existing peptide repositories such as PeptideAtlas [39], and should thereby expedite efforts to quantify of particular proteins of interest in the *Drosophila* heart by MRM.

In summary, the present study provides the first demonstration that proteomic studies are possible in *Drosophila* hearts. Though the extent of proteome coverage lags that of the well-studied mouse heart (4906 proteins) [33], through a combination of extensive dissection (100 *Drosophila* hearts can be harvested in a day) and careful data validation, we have compiled 1228 protein clusters from an organ whose mass is about 1/10^6^ that of mouse heart. Ongoing efforts using new strategies and instrumentation platforms will seek to extend peptidome/proteome coverage while reducing the number of hearts required as we lay the framework for quantitative protein-network approaches [40], [41] to study cardiomyopathy in *Drosophila*.

## Supporting Information

Table S1 **Proteins & Peptides** Panel 1: Read Me covers caveats associated with using these table(s) Panel 2: List of 1228 protein clusters, with identification probability and other buttressing peptide information. Panel 3: List of all peptides identified and associated with given protein isoform or protein cluster. Panel 4: List of all unique (non-redundant) peptides identified in this study. Panel 5: List of human homologues of identified *Drosophila* protein clusters. Homologues were found using batch searches of Homologene and Ensemble Compara databases. Panel 6: Distribution of spectra among the 4 technical replicates associated with the identified proteins.
(XLSX)

Table S2 **Assigned Spectra** Panel 1. Read Me covers caveats associated with using these table(s). Panel 2: List of each spectrum file assigned to a given peptide.
(XLSX)

Table S3 **Spectra ST Matches** All proteins identified on the basis of a single peptide, regardless of the number of assigned spectra, were cross-referenced against a reference *Drosophila* peptide dataset as described in the Methods section and Methods S1. Panel 1: The specific criteria for inclusion in these tables are presented. All other panels: Output from Spectra ST searches showing matches between our query spectra and the reference spectra.
(XLSX)

Table S4 **Spectra ST Matches** All proteins identified on the basis of a single peptide, regardless of the number of assigned spectra, were cross-referenced against a reference *Drosophila* peptide dataset as described in the Methods section and Methods S1. Panel 1: The specific criteria for inclusion in these tables are presented. All other panels: Output from Spectra ST searches showing matches between our query spectra and the reference spectra.
(XLSX)

Table S5 **Spectra ST Matches** All proteins identified on the basis of a single peptide, regardless of the number of assigned spectra, were cross-referenced against a reference *Drosophila* peptide dataset as described in the Methods section and Methods S1. Panel 1: The specific criteria for inclusion in these tables are presented. All other panels: Output from Spectra ST searches showing matches between our query spectra and the reference spectra.
(XLSX)

Table S6 **Spectra ST Matches** All proteins identified on the basis of a single peptide, regardless of the number of assigned spectra, were cross-referenced against a reference *Drosophila* peptide dataset as described in the Methods section and Methods S1. Panel 1: The specific criteria for inclusion in these tables are presented. All other panels: Output from Spectra ST searches showing matches between our query spectra and the reference spectra.
(XLSX)

Table S7 **Spectra ST Matches** All proteins identified on the basis of a single peptide, regardless of the number of assigned spectra, were cross-referenced against a reference *Drosophila* peptide dataset as described in the Methods section and Methods S1. Panel 1: The specific criteria for inclusion in these tables are presented. All other panels: Output from Spectra ST searches showing matches between our query spectra and the reference spectra.
(XLSX)

Table S8 **Spectra ST Matches** All proteins identified on the basis of a single peptide, regardless of the number of assigned spectra, were cross-referenced against a reference *Drosophila* peptide dataset as described in the Methods section and Methods S1. Panel 1: The specific criteria for inclusion in these tables are presented. All other panels: Output from Spectra ST searches showing matches between our query spectra and the reference spectra.
(XLSX)

Table S9 **High-Quality Spectra** Proteins identified on the basis of high quality spectra, as defined in the Methods section and Methods S1, but for which no match could be found using Spectra ST. Panel 1. The specific criteria for inclusion in these tables are presented. All other panels: High-quality spectra are presented, along with the explicit attributes of the spectra that conform to established CID-induced fragmentation biases.
(XLSX)

Table S10 **Proteins Absent from the Dataset of Brunner** ***et al*** **.** Panel 1: Protein isoforms and clusters were mapped to their CG identifiers and screened against the dataset of Brunner *et al*. [24]. Overlap is designated with “1” in column C; proteins unique to our study are designated “0”. Panels 2–4: Analysis of the proteins unique to our dataset with respect to the three branches of gene-ontology. Panel 2: Biological Processes. Panel 3. Cellular Components. Panel 4. Molecular Functions.
(XLSX)

Table S11 **Relative Protein Abundance** List of proteins that comprise the functional classes depicted in Figure 3A. These 245 proteins represent the most abundant proteins, comprising 20% of the identified protein isoforms or clusters and nearly 80% of all assigned spectra.
(XLSX)

Table S12 **Functional Annotation and Enrichment** Panel 1: Read Me covers caveats associated with using these table(s) Panel 2: Functional classification of identified *Drosophila* heart proteins using DAVID as described in the Methods section and Methods S1, in a sortable format. Panel 3: Sorted by enrichment score. Panel 4: Complete GO annotation for identified proteins.
(XLSX)

Table S13 **Cardiac Proteins Essential for Fly Survival, Orthologs of Vertebrate Proteins Critical for Heart Function, Pfam Analysis of** ***Drosophila*** **and Mouse Heart Proteomes and Proteins of Interest** The *Drosophila* cardiac proteome was compared with the work of Neely *et al.* [14] 74 identified proteins overlap with the 498 cardiac genes deemed essential for fly survival. Panel 1: Mapping the human and mouse orthologs of these proteins. Information includes the tissue distribution, disease-association and functional classification of these orthologues. Panel 2: GO annotation of the 74 overlapping proteins. Panel 3: Graphical representation of the preponderance of functional classes represented by the 74 overlapping proteins. Panel 4: Pfam domains represented in the *Drosophila* cardiac dataset. Panel 5: Pfam domains represented in the Mouse heart dataset of Bousette *et al.* [33]. Panel 6: Proteins in the *Drosophila* cardiac dataset with multiple isoforms. Panel 7. Listing of the major myofilament proteins identified. Panel 8: Proteins of interest with respect to mathematical models of cardiac function.
(XLSX)

Table S14 ***Drosophila*** **Cardiac Peptidome** Panel 1: Proteotypic peptides (as defined in the text) that unambiguously identify a specific protein isoform. Panel 2. Information-rich peptides (defined in text) that can be used to identify multiple protein isoforms. Panel 3. PeptideClassifier analysis of all 5169 unique (non-redundant) peptides.
(XLSX)

Methods S1 This supplement contains detailed description of experimental methods and apparatus.
(DOCX)
