# Supplementary material for: A Mighty Small Heart: The Cardiac Proteome of Adult Drosophila melanogaster
Source: PLoS One. 2011 Apr 25;6(4):e18497. doi: 10.1371/journal.pone.0018497 (PMC3081823; doi:10.1371/journal.pone.0018497)
Supplement: Methods S1 — This supplement contains detailed description of experimental methods and apparatus. (DOCX) [file pone.0018497.s015.docx]

Methods Supplement S1 - Table of Contents

Dissection of Drosophila hearts2

Fluorescence and Electron Microscopy of Drosophila Hearts 2

Sample Preparation, Fractionation and Digestion 2

HPLC and Mass Spectrometry: Instrumentation and Parameters 3

HPLC Parameters 3

Mass Spectrometer Settings3

Database Searching 4

Criteria for Protein Identification 4

Scaffold Thresholds 4

Screen for Poor Fragmentation4

Spectral Matching Against Reference Spectra 4

Manual Evaluation5

Protein Annotation and Classification6

Comparison of Drosophila cardiac tube dataset (Cammarato et al.) with the Extended Drosophila Proteome (Brunner et al.)6

1. Comparison of Protein Sequence Databases used for Proteomic Studies6

2. Comparison of Distinct Peptide Identifications 7

3. PeptideClassifier Analysis7

4. Proteotypic Peptide List8

5. Protein Overlap8

6. Final note on comparisons of our dataset with the extensive *Drosophila* proteome

of Brunner *et al.* 8

Comparison of Drosophila Cardiac Tube Proteomics Data (Cammarato et al.) with Transcriptomics data from Drosophila Hearts (Zeitouni et al.)9

Comparison with Genome-wide RNAi screen for essential cardiac genes (Neeley et al.)9

Comparison of Drosophila Cardiac Tube with Mouse Heart Proteome (Bousette et al.) 9

Caveats & Limitations Associated with the Application of Enrichment Analysis to Proteomic Data & Comparison of Proteomic Datasets 10

On the Utility of Single-Peptide and Single-Spectrum Matches11

On the Identification of Myofilament Protein Isoforms 11

References12

***Dissection of Drosophila hearts***

*yw* wild-type *Drosophila melanogaster* were raised on a standard yeast-agar medium at room temperature. The cardiac tubes of 145 male and female adult flies, ranging from 1 to 7 weeks of age, were dissected and exposed according to Vogler and Ocorr (2009)[[1](#_ENREF_1)]. Briefly, flies were anesthetized and the heads, ventral thoraces, and ventral abdominal cuticles were removed, exposing the heart tubes. All internal organs and abdominal fat were carefully removed leaving the heart and associated cardiac tissues. Dissections were performed under oxygenated artificial hemolymph at room temperature and all tubes were examined for activity prior to removal. The conical chambers (Fig 1) were grasped and the hearts were gently removed and quickly transferred to an Eppendorf tube containing 1.5 ml of artificial hemolymph on ice. The hearts continued to beat immediately following their removal. The tissue was pelleted (10,000 rpm) and washed three times quickly in distilled deionized water at 4°C. The sample was then lyophilized and the cardiac tubes dehydrated and stored at -80°C until digestion.

***Fluorescence and Electron Microscopy of Drosophila Hearts***

Fluorescence microscopy of TRITC-phalloidin labeled wild-type and myosin-GFP expressing *Drosophila* hearts was carried out as described in Alayari *et al*. (2009)[[2](#_ENREF_2)]. Fluorescent micrographs were acquired at 10 and 20X magnification. Electron microscopy was performed according to Wolf *et al.* (2006)[[3](#_ENREF_3)], however, prior to fixation the cardiac tubes were exposed and dissected free of extraneous debris as described by Vogler and Ocorr (2009)[[1](#_ENREF_1)]. Electron micrographs of thin sections through the conical chamber were acquired at 3,800X and 10,500X magnification.

***Sample Preparation, Fractionation and Digestion***

Dehydrated *Drosophila* hearts (145) were rehydrated in reducing SDS sample buffer supplemented with 6 M urea. Hearts were homogenized with a plastic homogenizer in an Eppendorf tube in a total volume of 45 µL, with care to minimize sample frothing. Samples were centrifuged for 5 min at 16000 x g to pellet particulates. 30 μL of the supernatant were loaded onto a 4-12% precast NUPAGE gel and electrophoresed for 35 min at 200 V. The gel was stained with Simply Blue (Invitrogen), and subsequently destained according to manufacturer’s instructions. 13 Gel tranches were excised with a razor blade, cut into 1x1x1mm pieces and subjected to trypsinolysis (sequencing grade modified trypsin; Promega), reduction and alkylation with iodoacetamide as described by Shevchenko *et al.*[[4](#_ENREF_4),[5](#_ENREF_5)].

***HPLC and Mass Spectrometry: Instrumentation and Parameters***

Protein identification by liquid chromatography-tandem mass spectrometry (LC-MS/MS) analysis of peptides was performed using an LTQ ion trap mass spectrometer (Thermo Fisher Scientific) interfaced with a nano 2DLC system (Eksigent, [www.eksigent.com](http://www.eksigent.com)).

**HPLC Parameters**

HPLC: Eksigent nano-2DLC pump

Injection: 8 µl;

Column: C18 75 µm column hand packed with YMC ODS-AQ 5 µm particle size, 120 angstrom pore size

Trap: C18 75 µM fused silica fritted with Kasil 1624 and hand packed to 3 cm with YMC 5-10 µM irregular C18

Buffer A: 0.1% formic acid

Buffer B: 0.1% formic acid /90% acetonitrile

Gradient:

1. Inject at 1% B from autosampler into nanoflowpath at 8.5% B.

2. Ramp up to 30% B in 15 minutes.

3. Ramp to 60% B by 18 minutes.

4. Ramp to 100% B by 22 minutes.

5. Hold for 2 minutes.

6. Return to 100 % A ending at 30 minutes.

Flow rate: 300 nanoliters per minute direct splitless

Quick gradients to 100% B twice are run in between every run to reduce carryover between samples.

**Mass Spectrometer Settings (LTQ)**

Mass Spectrometer: LTQ (ThermoFinnigan)

Spray emitter: 10 μm emitter (New Objective)

Spray Voltage: 2.4 kV

Scan Events: Precursor scans from 350-1800 m/z. Top 8 ions picked for MS/MS scans

Collision Energy: 30, (Q=0.250; Activation Time=30)

Dynamic Exclusion: repeat count 1

Exclusion duration: 20 seconds

Tune Method: Angiotensin_649_2.4kV

Following the first round of mass spectrometry, base peak chromatograms were inspected, and where warranted, loading was increased to maximize sensitivity and fully exploit the dynamic range of the mass spectrometer. To increase proteome coverage and to partially overcome the stochastic under-sampling typical of LC-MS/MS analysis[[6](#_ENREF_6)], three additional MS-runs were carried out for each gel tranche peptide extract, for a total of 52 LC-MS/MS runs.

***Database Searching***

Tandem mass spectra were extracted by Bioworks 3.3. All MS/MS samples were analyzed using Mascot (Matrix Science, London, UK; version Mascot) and X!Tandem (www.thegpm.org; version 2007.01.01.1). Mascot was set up to search a database of *D. melanogaster* reference sequences (Refseq) downloaded from the National Center for Biotechnology Information (NCBI) in FASTA format. The database was current as of 09/24/2008 and contained 20735 entries. X!Tandem searches were conducted using the same database. Searches were conducted using trypsin as the digesting enzyme. Mascot and X!Tandem were searched with a fragment-ion mass tolerance of 0.80 Da and a parent-ion tolerance of 1.5 Da. Carbamidomethylation of cysteine was specified in Mascot and X!Tandem as a fixed modification. Oxidation of methionine was allowed as a variable modification.

To successfully merge all Mascot and X!Tandem data in “Mudpit Mode” (approx 1 million spectra initially), the following workflow was adopted. Data derived from each gel piece was searched individually with Mascot version 2.0 (Matrix Science). Files (.dat) were loaded, uncompressed, into Scaffold 2.2.03 where the data from each file was searched with the bundled version of X!Tandem in “Unrestricted” mode such that the second search was also conducted against the full *D. melanogaster* Refseq database. Merging of all Mascot and X!Tandem data was accomplished by creating a new Scaffold session in which all Mascot (.dat) and X!Tandem (.xml) were files loaded in “Compressed Mode” and “Mudpit Mode”.

***Criteria for Protein Identification***

Scaffold (version 2.02.04; Proteome Software Inc., Portland, OR) was used to validate MS/MS based peptide and protein identifications. Peptide identifications were provisionally accepted if they had a >90.0% probability, as specified by Scaffold’s implementation of the Peptide Prophet algorithm[[7](#_ENREF_7)]. Proteins that contained similar peptides and could not be differentiated based on MS/MS analysis alone were grouped to satisfy the principles of parsimony[[8](#_ENREF_8)]. To maximize the sensitivity of discovery, given limited starting material (145 hearts, ≈20 μg of protein), identifications were accepted provisionally if they contained at least 1 statistically-validated unique peptide from 1 assigned spectrum. Recent studies have demonstrated the value of single hit protein identifications[[9](#_ENREF_9),[10](#_ENREF_10)], as long as care is taken to remove potential false positive identifications. False-discovery was minimized by subjecting these single-peptide identifications to the following **stringent** three-step evaluation process.

1. **Screen for poor fragmentation:** First, the list of candidate proteins was scanned for poor MS/MS spectra, resulting from poor peptide fragmentation, for which there were no consecutive b- or y-ion matches to peptide sequence. Occasionally these spectra are scored well by the search algorithms and are therefore assigned high confidence. Proteins identified solely on the basis of these spectra were removed.
2. **Spectral Matching:** Next, all remaining proteins identified on the basis of a unique peptide match, by either Mascot or X!Tandem, were cross-referenced by spectral matching. Specifically, peaklists from the best-scoring spectrum for each protein were submitted using the Spectra ST web application[[11](#_ENREF_11)] at the PeptideAtlas website[[12](#_ENREF_12),[13](#_ENREF_13)]. The query spectra data were searched against reference spectra from the *Drosophila melanogaster* peptide data set[[14](#_ENREF_14)] at the National Institute of Standards and Technologies (NIST). Queries matching reference spectra are documented in Tables S3-S8.
3. **Manual Inspection:** Finally, several high-scoring, high-quality, unique-peptide identifications did not match known reference spectra at PeptideAtlas/NIST. These protein candidates were inspected to assess the degree to which their spectra conformed to well-characterized fragmentation biases of certain amino acids, upon collision induced dissociation (CID). The criteria for the manual evaluation of MS/MS spectra have been summarized recently by Tabb *et al*. [[15](#_ENREF_15)]. Proteins meeting these criteria, noted explicitly, are documented in Table S9.

To recap, 1520 protein candidates initially met the minimal statistical threshold for provisional acceptance (one peptide with >90% probability). Our three-step evaluation removed 292 single-hit protein candidates. The final protein complement presented here contains 1228 proteins clusters (Tables S1, S2) identified by 5169 peptide matches from 29862 assigned spectra. Of these proteins, 462 were identified by a unique peptide (often many spectra). 341 of these 462 were validated by spectral matching of our query data against reference spectra[[11](#_ENREF_11),[16](#_ENREF_16)] from a comprehensive *Drosophila* dataset [[14](#_ENREF_14)] curated by NIST (see Tables S3-S8). The remaining 121 single-peptide identifications had assigned spectra that conform to known MS/MS fragmentation biases[[15](#_ENREF_15)], documented explicitly in Table S9.

From the information above we can provide a rough estimate of the false discovery rate at the protein level. The choice of 90% peptide confidence and 50% protein confidence according to Peptide Prophet and Protein Prophet algorithms respectively, resulted in 1520 protein candidates, from which 292 failed to meet reasonable criteria for a peptide match in the subsequent curation. Assuming that these 292 proteins represent false positive identifications, this would correspond to a false discovery rate at the protein level of ~19% prior to curation (which is consistent with reported results[[9](#_ENREF_9)]). The rate of false discovery is much lower among the 766 proteins identified by at least two peptides (90% confidence) and final protein confidence of 99.9% by Protein Prophet. In addition, the number of false positives remaining among proteins identified by at least one peptide at 90% confidence and subsequently cross-referenced against the spectral repository at NIST should be very low. Taken together, we estimate the protein level FDR to be in the low single digit percentage range for the final curated list of 1228 protein clusters.

The dataset will be deposited at a public proteomic data repository in accordance with journal guidelines.

***Protein Annotation and Classification***

Protein annotation and subsequent enrichment analysis was conducted with either the "Functional Annotation Table" tool at the DAVID Knowledgebase (http://david.abcc.ncifcrf.gov/)[[17](#_ENREF_17),[18](#_ENREF_18)] or using ProteinCenter (Proxeon). To use DAVID, the NCBI gi accession numbers of proteins identified by Scaffold were mapped to their corresponding UniProtKB accessions and/or Flybase gene numbers using ID Mapper at UniProtKB (www.uniprot.org).

Clustering and enrichment analysis was conducted using the "Gene Functional Classification Tool" at the DAVID Knowledgebase. From submitted gene lists, clusters are assembled on the basis of shared functional annotation terms (up to 75000) from 14 databases.

Analysis was conducted at medium stringency using default clustering parameters (i.e. minimum of 4 proteins per group). This served to minimize the number of excluded protein entries while keeping the number of clusters manageable.

Co-functioning (clustered) genes are enriched if their specific gene-annotation terms appear with greater frequency in the submitted list than they do in a reference or background genome (in this case *D. melanogaster*). The statistical significance of the enrichment (p-value) is assessed with a modified Fisher’s exact test. The final Enrichment Score, in turn, is the hypergeometric mean of all p-values presented on a negative logarithmic scale[[17](#_ENREF_17)]. Enrichment was conducted either using DAVID (Table S11) or ProteinCenter (Tables S10 and S12).

***Comparison of Drosophila cardiac tube dataset (Cammarato et al.) with the Drosophila proteome of Brunner et al[***[***14***](#_ENREF_14)***].***

**1. Comparison of Protein Sequence Databases used for Proteomic Studies**

|  | **Brunner et al., 2007** | **Cammarato et al.**  **2010** |
| --- | --- | --- |
| **Protein database** | BDGP v3.2 | NCBI RefSeq |
| Total protein sequences | 19177 | 20735 |
| Distinct protein sequences | 16743 | 17893 |
| Protein-coding gene models | 13792 | 14144 |
|  |  |  |
| ***In silico* proteotypic/information-rich**  **peptide analysis** |  |  |
| Tryptic peptides in range* | 382687 | 391328 |
| peptides common to both databases | 377553 | 377553 |
| peptides only present in BDGP3.2 | 5134 |  |
| peptides only present in NCBI RefSeq |  | 13775 |

* conducted using the program digestDB (part of the transproteomic Pipeline, TPP) as in Qeli & Ahrens[[19](#_ENREF_19)] , i.e., we considered only fully tryptic peptides of length 6 amino acids and above with a mass/charge ratio between 450 and 4500 Da (the analysis in Brunner *et al.* considered peptides of 6-55 amino acids)

**2. Comparison of distinct peptide identifications**

A comparison of the experimentally-identified peptide sequences revealed that, of the 5169 unique peptides (Table S1) 1293 were not found in the dataset of Brunner *et al.* and are, therefore, novel to the heart tube proteome. Importantly, only 25 peptides of the 5169 peptides matches found from searching the RefSeq database were not in BDGP3.2. Therefore the bulk of the novel identified peptides do not arise simply from the use of different databases for analysis, but rather, stem from the use of isolated *Drosophila* cardiac tubes.

**3. PeptideClassifier analysis**

The PeptideClassifier analysis, devised by Qeli and Ahrens[[19](#_ENREF_19)], is used to delineate the relationship between peptides , protein sequences (and identifiers) and their encoding gene-models. This classification will aid future targeted quantitative proteomic studies based on technologies such as multiple reaction monitoring (MRM) by revealing which peptides best distinguish protein isoforms. To provide the necessary clear relationship between gene model and protein identifiers, Refseq GI accession numbers were mapped to their corresponding CG protein identifiers. For 13 protein sequences, we manually added the CG identifier after blasting the respective protein sequence at Flybase.

By definition:

Class 1a peptides uniquely identify one protein sequence/isoform and its corresponding gene model.

Class 1b peptides uniquely identify one protein sequence which, however, can be the product of one of several transcripts encoded by one gene model that are alternatively spliced in either the 5' or 3' untranslated regions.

Class 2a peptides identify a subset of all protein isoforms encoded by a gene model.

Class 2b peptides are common to all protein isoforms encoded by a gene model.

Class 3a peptides identify a unique protein sequence encoded by multiple distinct gene models.

Class 3b peptides are sequences that are common to (often) unrelated proteins, and provide little information.

**The distribution of these types of peptides within the databases used by Brunner *et al.* and in our study (Cammarato *et al.*) were as follows.**

| **Class** | **Protein Sequence** | **Protein isoform(s)** | **Gene(s)** | **BDGP v3.2** | **NCBI RefSeq** |
| --- | --- | --- | --- | --- | --- |
| **1a** | **Unambiguous** | **Unambiguous** | **Unambiguous** | **283469** | **275231** |
| **1b** | **Unambiguous** | **Ambiguous** | **Unambiguous** | **30527** | **31698** |
| **2a** | **Ambiguous** | **Ambiguous** | **Unambiguous** | **8065** | **12216** |
| **2b** | **Ambiguous** | **Ambiguous** | **Unambiguous** | **56353** | **67597** |
| **3a** | **Unambiguous** | **Ambiguous** | **Ambiguous** | **234** | **210** |
| **3b** | **Ambiguous** | **Ambiguous** | **Ambiguous** | **4039** | **4376** |

**4. Proteotypic Peptide List**

Of all the tryptic peptides theoretically observable, only a fraction has physicochemical properties that favor detection by mass spectrometry. Fewer still, lend themselves to unambiguous protein identification. For example, class 3b tryptic peptides may arise from multiple unrelated proteins and therefore provide little information about the protein complement of a sample. Therefore, in Table S14, we present a list of observable peptides that identify specific protein isoforms (classes 1a, 1b and 3a; Panel A) or multiple protein isoforms encoded by the same gene model (class 2a and 2b; Panel B). Type 3b peptides are omitted. We also note which of these proteotypic, or information-rich, peptides were observed previously by Brunner *et al*. and which are novel to our study.

**5. Protein overlap**

The proteome study of Brunner *et al.* identified 9124 protein clusters whereas our study identified 1228 clusters. Dataset comparisons at the protein level are complicated by the fact that different databases were used for each study. By this analysis, 237 protein clusters (approx 19% of our proteome) were considered novel. See Table S10.

**6. Final note on comparisons of our dataset with the extensive *Drosophila* proteome of Brunner *et al.***

With respect to proteoypic peptides, it is important to assess whether a proteotypic peptide identified with a prior database version still is a prototypic peptide in the sense that it unambiguously identifies one protein sequence. Re-assessing the Brunner *et al.* data indicated that several of the proteotypic peptides listed at the time (identified by searches against BDGP3.2), when compared to the NCBI RefSeq database now can imply more than one protein sequence. Such peptides can, however, still provide valuable information, for example quantitative information for all isoforms encoded by a gene model. Such an approach is taken in the first phase of the human protein detection and quantification (hPDQ) project[[20](#_ENREF_20)].

***Comparison of Drosophila cardiac tube proteomics data (Cammarato et al.) with transcriptomics data from Drosophila hearts***

In order to assess the quality of our dissection protocol, we screened the literature for a transcriptomics study of *Drosophila* heart tubes. The closest matching dataset of high quality that we could find was a time course study of early heart development, where the cardiac tubes had been carefully dissected at eight different time points after pupae formation (21 hours to 48 hours APF), and 4 replicates had been analyzed for each time point. The data were kindly provided by Dr. Laurent Perrin (Developmental Biology Institute Marseille Luminy).

To be able to compare transcriptomics and proteomics datasets, we first mapped the primary Flybase gene identifiers of the probe sets (FBgn nomenclature) of the International *Drosophila* Array Consortium oligonucleotide microarray to primary Flybase CG gene identifiers. 4677 distinct CG gene model identifiers could be matched among the 4853 genes that were consistently expressed above a threshold value (in the original study defined as two times the average expression level of negative controls). We then assessed the overlap among 1207 gene models that encode the 1228 proteins identified in our study with the gene models consistently expressed in the transcriptomics study: for 854 of these (71%), we also recorded proteomics evidence for their expression. Furthermore, for another 285 gene models (24%) we found evidence that their protein products are expressed in adult heart, while their corresponding transcripts were not consistently expressed above the chosen threshold during early stages of heart development.

We would like to note that this qualitative comparison is an approximation due to several reasons: i) the probes on the array (FBgn annotation) where mapped against the Flybase CG annotation identifiers from 2006 which gave the best overlap, ii) while the array contains splice isoform specific probes, we only consider the gene model level (we took the first gene model identifier if more than one gene model could be implied by our peptide evidence), iii) we compare a transcriptomics dataset from a time-course on pupal heart development to a proteomics dataset from adult hearts. Despite these limitations, we do find a large overlap between these datasets.

***Comparison with genome-wide RNAi screen for essential cardiac genes (Neeley et al.)***

We recently performed a genome-wide RNAi screen to identify conserved cardiac genes whose products are essential for *Drosophila* survival under conditions of stress[[21](#_ENREF_21)]. Heart-restricted silencing of 498 genes significantly increased mortality when the flies were exposed to elevated temperatures. Of the 1228 cardiac proteins identified in the current study, 74 were shown to be indispensable via the RNAi screen (Table S13). Thus, these 74 proteins are vital for survival and are abundant enough to be positively identified via our proteomic approach. Accordingly, ~73% of the orthologues show associations (determined via the NextBio web-based platform (<http://www.nextbio.com/b/nextbio.nb>)) with the cardiovascular system of vertebrates (humans and/or mice) and ~40% associate with diverse cardiac related disorders including cardiomyopathy, myocardial infarction, cardiac arrest and heart failure.

***Comparison of Drosophila Cardiac Tube with the Mouse Heart Proteome (Bousette et al.)***

Pfam domains present within mouse and *Drosophila* heart proteomes (extracted using ProteinCenter (Proxeon)) were mapped to Gene Ontology (GO) terms using the "pfam2go" mapping platform (dated 2009/10/01) provided by the GO consortium [[22](#_ENREF_22),[23](#_ENREF_23)]. GO term enrichment analysis was performed via Ontologizer 2.0, a multifunctional software tool for GO term enrichment analysis and data exploration [[24](#_ENREF_24)], with the Topology-Elim algorithm [[25](#_ENREF_25)] which integrates the graph structure of the GO in testing for group enrichment, and Bonferroni correction to address the problem of multiple comparisons. Enrichments were calculated relative to the functions of all Pfam domains present in the respective genome, as determined by hmmscan from the HMMER 3.0 package (http://hmmer.janelia.org/) using HMMs from Pfam 24.0 [[26](#_ENREF_26)] with the “gathering” (GA) cutoff scores provided by Pfam. Both the mouse and *Drosophila* protein predictions were downloaded from the Ensembl database, release 56 (<ftp://ftp.ensembl.org/pub/current_fasta/mus_musculus/pep/>, <ftp://ftp.ensembl.org/pub/current_fasta/drosophila_melanogaster/pep/>).

***Caveats & Limitations Associated with the Application of Enrichment Analysis to Proteomic Data & Comparison of Proteomic Datasets***

Enrichment analysis provides a useful method for identifying gene-classes of interest owing to their over-representation relative to a genomic background. For the analysis of proteomic data, however, caution should be exercised, as overrepresentation of protein classes conceivably may stem from physicochemical properties (size, amino acid composition, solubility) that favor robust identification. Conversely, other protein classes are frequently recalcitrant to proteomic analysis, owing to low abundance or hydrophobicity, and likewise confound functional enrichment analysis. For instance, we did identify several ion-handling proteins (channels, pumps and exchangers) in the excitable *Drosophila* heart, yet with lower frequency than found in the genome. Consequently, ion-handling proteins were not highlighted as significantly enriched.

Functional bioinformatic comparisons of proteomic datasets also entail limitations. We illustrated the true heart-like character of the *Drosophila* cardiac tube by comparing the ontologies and Pfam domains between the *Drosophila* and mouse datasets. To the extent that major protein classes have been identified and are conserved, this supports our hypothesis. Differences between the datasets are more difficult to interpret. They may stem from true biological differences between the two species. However, there are sufficient differences in methodology, instrumentation and analysis between this study and the work by Bousette *et al.*[[27](#_ENREF_27)] that failure to find correlates among protein families may arise for technical rather than biological reasons.

***On the Utility of Single-Peptide and Single-Spectrum Matches***

In this study, we have included proteins identified on the basis of a single peptide (468; 38% of total proteins reported) of which about half (228, 19% of proteins reported) were assigned on the basis of a single spectrum/peptide match. In the latter case, the spectra were, therefore, found in only 1 of 4 replicate MS runs, in accordance with well-documented stochastic undersampling of low abundance proteins in shotgun proteomics experiments. Inclusion of single-spectrum matches can greatly extend the depth of the proteome coverage provided they are carefully screened and validated to minimize false discovery (see p. 4 & 5 above). It could be argued, however, that such identifications have limited prospective value for quantitative MRM strategies and that only peptides present in each replicate are likely to serve as a meaningful MRM resource.

Yet developing extended observable peptide maps of tissues is likely one of the best prospects for obtaining near-complete proteomes in the future. Considering only peptides found in all technical replicates limits future quantitative studies to the most abundant proteins, or else it implies that there is sufficient source tissue to fractionate samples repeatedly such that rare proteins are identified in all replicates. One of the aims of this study was to develop proteomic resources what will allow us to study mechanisms of cardiac pathogenesis in the *Drosophila* model system using a systems biology approach. Clearly, the small size of the *Drosophila* heart represents a challenge, as gathering sufficient protein yields is highly labor-intensive. Moreover, extensive subfractionation of these hearts is impractical at present.

If we are ever to achieve a full *Drosophila* cardiac proteome, it is therefore imperative to consider all solid (well-curated) peptide evidence, regardless of how often it is observed by data-dependent acquisition in shotgun proteomics experiments. Moreover, it does not follow that infrequently-observed peptides are ill-suited for MRM study design, since this technique can be 10-100 fold more sensitive than shotgun approaches.

To be sure, protein quantitation by MRM on the basis of a single peptide is ill-advised. Therefore, the single-hit protein identifications presented here do not, by themselves, contain sufficient information for quantitative MRM. This *Drosophila* cardiac resource, including 1293 new peptides and 247 new proteins, should be used in conjunction with other resources, including peptide/protein repositories (e.g. PeptideAtlas) as well as tools for de novo proteotypic peptide prediction.

***On the Identification of Myofilament Protein Isoforms***

Of the identified proteins, 20 were identified with multiple distinguishable isoforms, many of which fell within the myofilament class (Table S13). Multiple isoforms of myosin heavy chain, troponin C, wings up (TnT), and upheld (TnI) were identified. The presence of various isoforms likely reflects the fact that the cardiac tube consists of several accessory structures (i.e. valves, ostia etc) and is attached to the body wall by fine suspensory fibrils, a poorly developed dorsal diaphragm and four pairs of alary muscles[[28](#_ENREF_28),[29](#_ENREF_29)]. A striated ventral longitudinal muscle layer, pericardial cells and sheets of fat cells are also intimately associated with the heart[[28](#_ENREF_28),[29](#_ENREF_29)].

**References**

1. Vogler G, Ocorr K (2009) Visualizing the beating heart in *Drosophila*. Journal of Visual Experiments.

2. Alayari NN, Vogler G, Taghli-Lamallem O, Ocorr K, Bodmer R, et al. (2009) Fluorescent labeling of *Drosophila* heart structures. Journal of Visual Experiments 13.

3. Wolf MJ, Amrein H, Izatt JA, Choma MA, Reedy MC, et al. (2006) *Drosophila* as a model for the identification of genes causing adult human heart disease. Proceedings of the National Academy of Sciences of the United States of America 103: 1394-1399.

4. Shevchenko A, Wilm M, Vorm O, Mann M (1996) Mass spectrometric sequencing of proteins silver-stained polyacrylamide gels. Analytical Chemistry 68: 850-858.

5. Shevchenko A, Tomas H, Havlis J, Olsen JV, Mann M (2007) In-gel digestion for mass spectrometric characterization of proteins and proteomes. Nature Protocols 1: 2856-2860.

6. Tabb DL, Vega-Montoto L, Rudnick PA, Variyath AM, Ham A-JL, et al. (2010) Repeatability and Reproducibility in Proteomic Identifications by Liquid Chromatography and Tandem Mass Spectrometry. Journal of Proteome Research 9: 761-776.

7. Keller A, Nesvizhskii AI, Kolker E, Aebersold R (2002) Empirical Statistical Model To Estimate the Accuracy of Peptide Identifications Made by MS/MS and Database Search. Analytical Chemistry 74: 5383-5392.

8. Nesvizhskii AI, Vitek O, Aebersold R (2007) Analysis and validation of proteomic data generated by tandem mass spectrometry. Nature Methods 4: 787-797.

9. Grobei MA, Qeli E, Brunner E, Rehrauer H, Zhang R, et al. (2009) Deterministic protein inference for shotgun proteomics data provides new insights into Arabidopsis pollen development and function. Genome Research 19: 1786-1800.

10. Gupta N, Pevzner PA (2009) False Discovery Rates of Protein Identifications: A Strike against the Two-Peptide Rule. Journal of Proteome Research 8: 4173-4181.

11. Lam H, Deutsch E, W. , Eddes JS, Eng JK, King N, et al. (2007) Development and validation of a spectral library searching method for peptide identification from MS/MS. PROTEOMICS 7: 655-667.

12. Loevenich S, Brunner E, King N, Deutsch E, Stein S, et al. (2009) The Drosophila melanogaster PeptideAtlas facilitates the use of peptide data for improved fly proteomics and genome annotation. BMC Bioinformatics 10: 59.

13. Desiere F, Deutsch EW, King NL, Nesvizhskii AI, Mallick P, et al. (2006) The PeptideAtlas project. Nucleic Acids Research 34: D655-658.

14. Brunner E, Ahrens CH, Mohanty S, Baetschmann H, Loevenich S, et al. (2007) A high-quality catalog of the *Drosophila* melanogaster proteome. Nature Biotechnology 25: 576-583.

15. Tabb DL, Friedman DB, Ham A-JL (2006) Verification of automated peptide identifications from proteomic tandem mass spectra. Nature Protocols 1: 2213-2222.

16. Lam H, Deutsch EW, Eddes JS, Eng JK, Stein SE, et al. (2008) Building consensus spectral libraries for peptide identification in proteomics. Nature Methods 5: 873-875.

17. Huang DW, Sherman BT, Lempicki RA (2008) Systematic and integrative analysis of large gene lists using DAVID bioinformatics resources. Nature Protocols 4: 44-57.

18. Dennis G, Sherman B, Hosack D, Yang J, Gao W, et al. (2003) DAVID: Database for Annotation, Visualization, and Integrated Discovery. Genome Biology 4: P3.

19. Qeli E, Ahrens CH (2010) PeptideClassifier for protein inference and targeted quantitative proteomics. Nature Biotechnology 28: 647-650.

20. Anderson NL, Anderson NG, Pearson TW, Borchers CH, Paulovich AG, et al. (2009) A Human Proteome Detection and Quantitation Project. Molecular & Cellular Proteomics 8: 883-886.

21. Neely GG, Kuba K, Cammarato A, Isobe K, Amann S, et al. (2010) A Global In Vivo *Drosophila* RNAi Screen Identifies NOT3 as a Conserved Regulator of Heart Function. Cell 141: 142-153.

22. Ashburner M, Ball C, Blake J, Botstein D, Butler H, et al. (2000) Gene Ontology: tool for the unification of biology. Nature Genetics 25: 25-29.

23. Hunter S, Apweiler R, Attwood T, Bairoch A, Bateman A, et al. (2009) InterPro: the integrative protein signature database. Nucleic Acids Research 37: D211-215.

24. Bauer S, Grossmann S, Vingron M, Robinson P (2008) Ontologizer 2.0--a multifunctional tool for GO term enrichment analysis and data exploration. Bioinformatics 24: 1650-1651.

25. Alexa A, Rahnenführer J, Lengauer T (2006) Improved scoring of functional groups from gene expression data by decorrelating GO graph structure. Bioinformatics (Oxford, England) 22: 1600-1607.

26. Finn RD, Mistry J, Tate J, Coggill P, Heger A, et al. (2010) The Pfam protein families database. Nucleic Acids Research 38: D211-222.

27. Bousette N, Kislinger T, Fong V, Isserlin R, Hewel JA, et al. (2009) Large-Scale Characterization and Analysis of the Murine Cardiac Proteome. Journal of Proteome Research 8: 1887-1901.

28. Miller A, editor (1950) *The internal anatomy and histology of the imago of Drosophila melanogaster*. New York: Wiley.

29. Rizki TM, Rizki RM (1978) Larval adipose tissue of homoeotic bithorax mutants of *Drosophila*. Developmental Biology 65: 476-482.
